# How external and internal resources influence user action: the case of infusion devices

**DOI:** 10.1007/s10111-016-0392-0

**Published:** 2016-09-21

**Authors:** Ioanna Iacovides, Ann Blandford, Anna Cox, Jonathan Back

**Affiliations:** 1 Institute of Educational Technology, The Open University, Walton Hall, Milton Keynes, MK76AA UK; 2 UCL Interaction Centre, University College London, 62-77 Gower Street, London, WC1E 6BT UK; 3 Centre for Applied Resilience in Healthcare, King’s College London, 57 Waterloo Road, London, SE1 8WA UK

**Keywords:** Human error, Conceptual fit, Healthcare, Qualitative research

## Abstract

Human error can have potentially devastating consequences in contexts such as healthcare, but there is a rarely a simple dichotomy between errors and correct behaviour. Furthermore, there has been little consideration of how the activities of users (erroneous and otherwise) relate to the conceptual fit between user and device, despite the fact that healthcare technologies are becoming increasingly prevalent and complex. In this article, we present a study in which nurses’ conceptions of infusion device practice were elicited to identify misfits. By focusing on key concepts that users work with when setting up infusions and the extent to which the system supports them, our analysis highlights how actions are influenced by the different resources available to users including: the device itself; supporting artefacts; the conceptual understanding of the user; and the community of practice the user is part of. The findings reveal the ways in which users are resourceful in their day-to-day activities and also suggest potential vulnerabilities within the wider system that could threaten patient safety. Our approach is able to make previously under-explored aspects of practice visible, thus enabling insight into how users act and why.

## Introduction

Human error remains a significant concern across safety–critical contexts, including healthcare. For instance, the Medicines and Healthcare Products Regulatory Agency in the UK attributed 21 % of incidents involving infusion devices to user error (MHRA 2013). Given the potentially severe consequences of errors, previous research has focused on incident reports to examine the circumstances surrounding errors that have occurred (e.g. Benner et al. 2002). However, adverse events are often under-reported (Husch et al. 2005; Morag et al. 2012), while the reports themselves often lack the necessary detail to establish why the error occurred (Nemeth et al. 2009a, b). In addition, Dekker (2007) argues that error counting systems “uphold an illusion of rationality and control but may offer neither real insight nor productive routes for improving safety”. In contrast, within this paper, we focus on investigating a device’s “fitness for purpose” (Blandford et al. 2014b) and on understanding how work is carried out in context in order to highlight system vulnerabilities that can be addressed to reduce the likelihood of error and improve patient safety.

As Norman (2002) points out, there is not “a simple dichotomy between errors and correct behavior” (p. 140) and there is much that happens in healthcare practice that is “invisible” (e.g. Furniss et al. 2011a). For instance, Furniss et al. (2011b) introduce the notion of “unremarkable errors” in healthcare that are described as “low-level disturbances” or “performance deviations” that occur during day-to-day activities: e.g. entering the wrong number into a device, then correcting it or forgetting to tell a patient how long their treatment will last. These minor deviations often remain invisible despite the fact that investigating why they occur, and how users try to recover from them, could have significant design implications on a technological and socio-technical level. Blandford et al. (2014a) argue that, without an understanding of actual use feeding into design, devices are often developed and implemented in ways that are not fit for purpose.

Assessing fitness for purpose requires further attention as technology is not only growing more prevalent in healthcare contexts but also becoming more complicated. For instance, Douglas and Leigh (2001) note that devices that were once only used in critical care units are now commonplace in general wards. Infusion devices are a case in point; these are used to administer intravenous (IV) drugs to patients after being programmed by clinical staff who input data into the pumps regarding the volume and rate (volume/time) of medication to be delivered. However, there is evidence that users sometimes confuse key concepts (such volume and rate; Garmer et al. 2002), suggesting that devices do not always support appropriate conceptual understandings. Meanwhile, technology continues to evolve with the introduction of “smart pumps” (which include software that requires further information about the patient and medication to be entered so it can perform additional checks to detect possible errors). However, the uptake of smart pump technology has been slow in the UK (Iacovides et al. 2014), and functions tend to be underused due to a lack of user understanding (Lamsdale et al. 2005; Rothschild et al. 2005; Nemeth et al. 2009a, b). Furthermore, there is evidence that the growing complexity of these devices is getting in the way of users forming mental models that reliably support device operation (Nunnally et al. 2004). In addition to having to manage demanding workloads, nursing staff are expected to be competent in using these increasingly complex devices, regardless of their clinical and technological expertise (Iacovides et al. 2013). However, while previous research has explored the use of technology through evaluating the general usability of medical devices such as infusion pumps (e.g. Graham et al. 2004), and focusing on specific design issues such as number entry input (e.g. Cauchi et al. 2014), very little attention has been paid to the mismatches that occur between user and device and how these may cause vulnerabilities within the wider system.

### Background

Existing usability methods such as heuristic evaluation, task analysis and think aloud have previously been applied to evaluate infusion devices (e.g. Zhang et al. 2003; Ginsburg 2005; Lamsdale et al. 2005), but these tend to focus on task structures rather than conceptual fit between user and device. While one of the heuristics that experts need to apply as part of a heuristic evaluation states that the “image of the system perceived by users should match the model the users have about the system” (p. 25; Zhang et al. 2003), questions remain about what models users actually have of infusion devices and how well a particular device is able to match them.

A focus on mismatches can yield insight into the causes of error within safety–critical domains. Baxter et al. (2007) note that cognitive mismatches can take many forms, including “mode confusion” where pilots believe that the aircraft system is in one mode when in fact it is operating in a different one. The authors suggest that problems can arise due to a lack of transparency in the automation’s interface, affecting a pilot’s ability to accurately predict the aircraft’s behaviour. However, within the context of healthcare, focus has been less on mismatch between user and device and more on mismatches between different people’s mental models; e.g. Morag et al. (2012) examined differences between the mental models of physicians and nurses on a gynaecology ward. While mismatches between people’s mental models of work processes can lead to difficulties and failures, there is a need to also consider how the technology used within a ward environment may also contribute to error when there is a mismatch between user and device. In addition, given that cognition can be distributed across people, technology, physical context and time (Hutchins 1995), there is need to understand the interaction not just between user and device but between user, device and the supporting artefacts e.g. prescription charts, personal notes etc. that are used in actual practice (Back and Cox 2013; Back et al. 2013).

Our approach draws upon previous work by Blandford and colleagues (e.g. Blandford et al. 2008; Blandford 2013), which considers the extent to which a system is able to support users’ conceptual understanding. In order to carry out an analysis of conceptual fit, the first step involves eliciting key user conceptions before identifying mismatches between user and device, i.e. misfits between “the way the user thinks and the representation implemented within the system” (p. 394; Blandford et al. 2008). For instance, after conducting a contextual inquiry within a law firm, Attfield and Blandford (2011) identified several issues within a current-awareness alert system that related to a lack of representation of key user concepts at the interface and system level e.g. each alert item contained information from particular articles or documents, which were often important for the user to access, but the system did not always support a direct link to the source material. This issue led users to stop interacting with the system and having to carry out separate online searches. However, there has been little work in the area of medical device safety that has investigated the extent to which a particular technology supports user understanding.

Given that people’s conceptual models are informed by the tools available to them and the context they work within, we aimed to understand how both internal and external resources influence user action (Hutchins 1995). Therefore, we focus on conceptions of infusion device practice, which relate to concepts pertaining to the device and those relating to the domain of use, to explore the interaction between user, device and supporting artefacts. The aim of this study was to identify these mismatches as a necessary step to informing future design.

## Method

### Overview

As Wolf et al. (2006; p. 5) “Understanding the complex nature of nurses’ cognitive work offers a new perspective for the analysis of the environmental conditions that create risk for medical errors or omissions in care”). While observation can yield useful insights into practice, the rationale behind particular actions can be unclear (Wolf et al. 2006). In addition, the demanding nature of nurses’ work means it can be distracting to ask a member of staff to explain aspects of practice while they are working in a busy environment (Furniss et al. 2014; Blandford et al. 2015). Due to similar reasons, methods such as contextual inquiry (Beyer and Holtzblatt 1998), involving observation and interviews that take place within a user’s normal work environment, can be difficult to implement in healthcare contexts. Thus, we looked to teach-back techniques that aim to elicit user conceptions through asking participants to carry out tasks on a device and explain what they are doing as if they were teaching a novice. For instance, Clark and Sasse (1997) compared groups of new and existing users of an online tool, which facilitated setting up and participating in online events, to teach a contrived co-learner about the tool after they had spent some time using it. Transcripts of the sessions were then analysed to elicit key user concepts regarding infusion device practice and to consider any mismatches that arose when the user was interacting with the device and any supporting artefacts.

Our study included two semi-structured interviews, where the first session was introductory, focusing on eliciting background information, and the second revolved around the teach-back tasks. A thematic analysis was then carried out on the transcripts (Braun and Clarke 2006) to identify particular mismatches relating to the interaction between user, device and supporting artefacts. The Research and Development team at the hospital reviewed the study and concluded it did not require National Health Service ethical approval. The study was granted ethical approval by the University Research Ethics Committee, while permission was sought from the Associate Dean of Nursing, who also helped to facilitate access to nursing staff.

### Participants

Seventeen nurses (16 female, 1 male; mean age: 39.6 years) took part in the study. This was a snowball sample recruited from across a number of clinical areas within a large, urban, acute UK hospital. Table 1 indicates which areas each nurse worked within, how many years of experience they had (mean = 11.71; standard deviation = 7.81) and how long they had worked at the hospital (mean = 4.94; standard deviation = 4.35). The majority of nurses involved in the study worked in oncology, Paediatrics and intensive treatment units (ITU) though one nurse worked on a surgical ward and three others worked in cancer research (e.g. delivering new drugs as part of clinical trials). Efforts to standardise infusion devices have led to many UK hospitals attempting to use a single brand of infusion device across the whole organisation (Iacovides et al. 2014), so all the nurses who participated in the study regularly used the same model of large volume infusion pump which had been used at the hospital for several years. We recruited nurses from different clinical areas and who had a range of experience in order to increase the generalisability of our findings. Out of the 17 participants recruited, 14 also completed the second interview, which took place 1–2 weeks after the first.

**Table 1:** Participant details

Participant	Clinical area	Years of nursing experience	Years at the hospital
A	Oncology	5	5
B	Oncology	19	1
C	Research nurse	6	2.5
*D*	*Oncology*	*9*	*1*
E	Research nurse	5.5	0.5
*F*	*Oncology*	*8*	*8*
*G*	*Paediatric*	*21*	*3*
H	Paediatric	1	1
K	Research nurse	23	7
L	Paediatric	11	9
M	Paediatric	4	1
N	Paediatric	5	0.5
O	Surgery	4	2
P	ITU	13.5	8.5
Q	ITU	24	8
R	ITU	20	14
S	ITU	20	12

Italicised participants only took part in the first interview

### Procedure

For the convenience of participants, interviews took place at meeting rooms on the hospital site. Two interviews were planned with each participant, and each interview was conducted by two interviewers; one led the interview and focused on device use, and the other asked questions about supporting artefacts.

In the first interview, participants were asked to give an overview of their main role and responsibilities, to explain what infusion device training they had received and to explain how they use infusion devices to treat patients. Participants were also asked about common and uncommon infusions and if they, or another staff member, had ever had any difficulties with the pump. To further understand the interaction between user, device and environment, this included a discussion of the different artefacts they used to support the delivering of IV medications e.g. prescription charts, handover sheets and any personal notes or calculations. The interview was audio recorded and lasted approximately 30 min.

For the second interview, participants were asked to bring in recent prescription charts containing IV prescriptions they had delivered through a pump and any additional supporting artefacts, such as personal notes, that they used as part of the delivery process (after having redacted any patient and clinician information with a black marker pen). At the start of the interview, each participant was asked to discuss the charts and any additional materials they brought in and explain how they were used to deliver particular infusions. The teach-back activity occurred in the second part of the interview, where a training infusion pump was available for participants to carry out two programming tasks. These tasks were selected from the real-world examples they had brought in e.g. a nurse from Paediatrics was asked to demonstrate how she would set up the pump to deliver IV fluids to a child suffering from dehydration. While carrying out the tasks, the interviewers prompted the participant to explain what they were doing and why, as if they were nurses who had completed their IV therapy and device training and has recently started on the ward. The prompts included questions about how the medications were prepared, the exact values that were entered into the device, checks that occurred before and after the infusion was running and what happened at the end of an infusion. The second session was video recorded and lasted between 45 and 60 min. Participants were paid £35 at the completion of both interviews.

#### Setting up an infusion device

Before discussing the main analysis and findings, we first provide a broad overview of how the infusion device is normally set up to deliver medication to illustrate how programming the pump is only part of the whole procedure. The process usually begins with a particular infusion being prescribed to a patient and via a prescription chart. The nurse then has to collect the infusion, or sometimes they have to make it up themselves by combining a particular drug with intravenous (IV) fluid. Depending on the medication prescribed, the units can be given as a weight (e.g. micrograms) or a volume (e.g. millilitres). Nurses often have to carry out different calculations, such as establishing what dose a patient requires (e.g. based on their weight) or converting prescribed units into values they can enter into the pump. The pump itself requires a volume, rate and/or time to be entered. Before programming the pump, the nurse is required to carry out a series of checks to make sure the right drug and dose is being administered to the right patient, through the right route, within the right time frame. Also, a second nurse is often asked to provide a second check of the treatment prepared or retrieved.

The nurse then sets up the infusion by inserting the giving set (the line connecting the bag or bottle of medication to the patient) into the pump and “priming” the line. Priming involves running the infusion through the giving set and ensuring there are no air bubbles in the line, and can be done manually or through the pump. The giving set also has a “roller clamp” attached to it (this is usually a plastic device equipped with a small roller that may be rolled to close off or open IV tubing). When closed, the roller clamp stops the liquid running out of the line, while it needs to be opened to allow the infusion to run through. After priming, the line is connected to the patient via a particular point of access, e.g. a “cannula” (a thin tube inserted into a vein, often in the back of the hand or the inside of the arm). The nurse then enters the required values into the device and starts the infusion. The nurse administering the infusion must then sign the patient’s medicine administration chart, which must also be signed by the second nurse to confirm the appropriate checks have occurred.

When the infusion is running, various alarms can go off to indicate issues such as air in the line (small air bubbles may build up over time), an occlusion (for instance, the patient may be lying on the tubing), the battery is running low, the infusion is about to finish or the infusion has ended. Depending on the type of infusion, patients may also need regular checks, e.g. to assess fluid intake and output or to make sure they are not having an adverse reaction. Particularly in an intensive care context, where patients are often receiving multiple drugs via different devices, vital signs such as blood pressure are monitored closely for any changes.

#### Analysis

All interview sessions were transcribed, and a thematic analysis was carried out using NVivo 10. The analysis focused on eliciting key user conceptions related to infusion device practice and mismatches between user and device i.e. misfits between “the way the user thinks and the representation implemented within the system” (p. 394; Blandford et al. 2008).

Early analysis focused on transcripts from both interviews, where key user concepts were elicited e.g. “infusion”, “giving set”, “priming” in order to facilitate consideration of how well the system was able to support them. Later stages then focused on the transcripts from the second interview, where themes that related to identifying different mismatches and how they could lead to error were identified and refined. The process involved investigating particular instances of confusion within the transcripts (e.g. around what an interface symbol represented or what details were checked once an infusion was set up and running) before examining the associated video footage to clarify the details referred to during teach-back. The themes were identified by iterating between the phases outlined by Braun and Clark (2006) before writing up the findings: familiarisation with the data; generating initial codes; searching for themes; reviewing themes; defining and naming themes. Blandford (2013) argues that inter-rater reliability is not an appropriate way to validate a rich interpretative data analysis; instead, the focus was on discussions between the authors regarding emerging themes and basing interpretations on triangulating interview transcripts with video recordings and relevant supporting documents (such as prescription charts and the device manual). For instance, one of the emerging themes related to the fact that nurses have to work with a range of units when delivering infusions—in addition to mentioning different units within the interviews, the documents supplied by nurses illustrated how medications differed in terms of the units they were prescribed in.

## Findings

The final set of themes are: the range of measurement units; interface symbols and options; configuration of the device; checking; and volume to be infused (VTBI) values.

### Range of measurement units

The infusion device works with three sets of values: volume to be infused (VTBI), represented in mls (millilitres); time represented in hours and minutes; and rate, represented in mls per hour. However, nurses have to work with a whole range of different units depending on the type of infusion to be delivered.

**Fig. 1:**
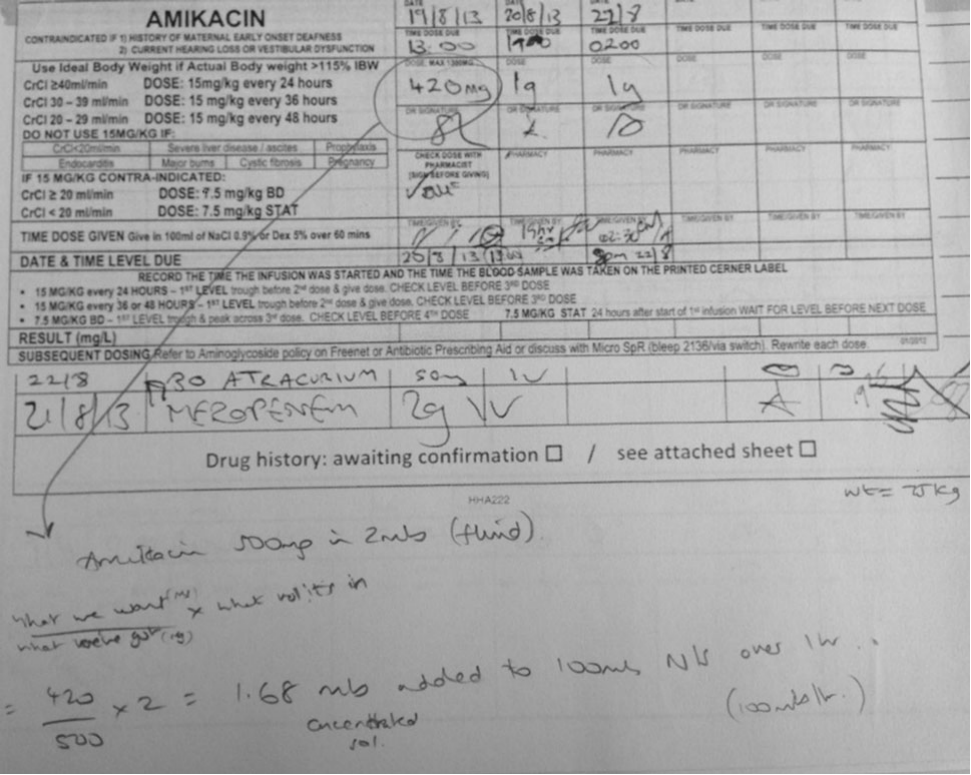
Prescription chart example

For instance, some therapies are not premixed by the hospital pharmacy and therefore require preparation prior to administration. This involves ensuring that the correct dose is added to the bag of fluid. Figure 1 illustrates a supporting artefact, that contains a calculation so that AMIKACIN can be infused at a rate of 100 ml/hr. The nurse had to use a formula to calculate how much AMIKACIN should be added to the bag of fluid. The manufacturer provides AMIKACIN in 500 mg doses in 2 ml of fluid. A calculation was used to determine that 1.68 ml of the 2 ml AMIKACIN fluid needed to be added to the bag since the prescription was for 420 mg.

**Fig. 2:**
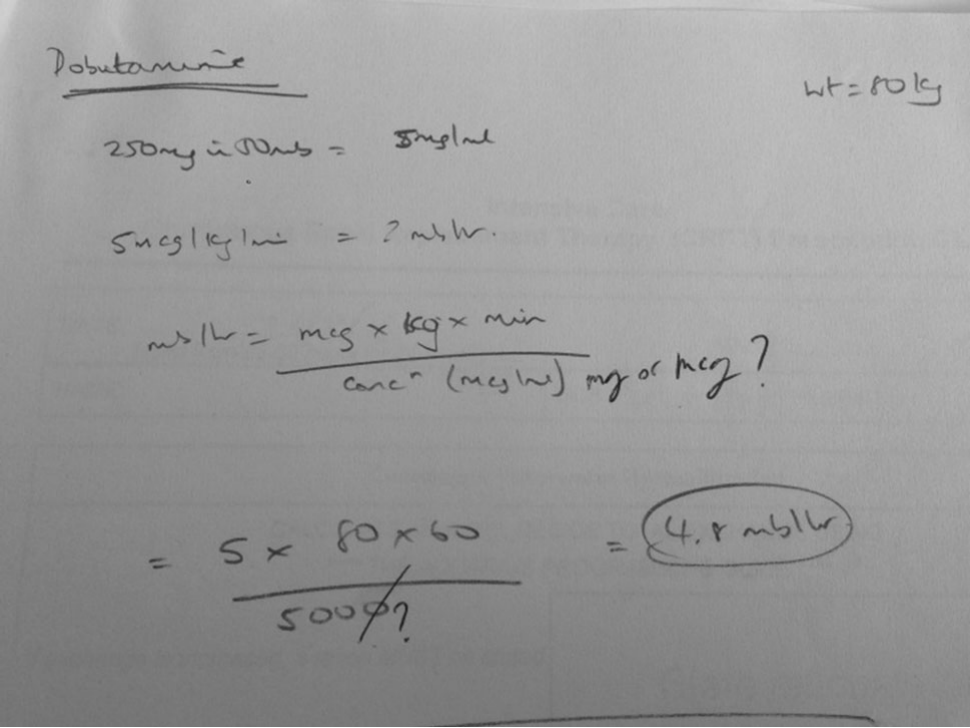
Calculation example

Another example of when calculations occur is in therapies where patient weight needs to be factored into establishing the correct dose. Figure 2 illustrates an additional supporting artefact, where the infusion rate for DOBUTAMINE was calculated, through the use of a formula. Firstly, the dose of the medication per ml has to be determined. In this case there is 5 mg per ml. The next stage involves multiplying the derived dose per ml by the patient’s weight and the number of minutes in 1 h. This is then divided by the drug concentration. Sometimes this drug concentration is in micrograms rather than milligrams which requires units to be converted. The infusion rate per hour can then be calculated; in this case it is 4.8 ml/hr.

Thus, the units on the prescription chart do not always correspond directly to what values need to be entered into the pump, creating potential for error. While recent developments in smart pump technology can allow users to work with dose measurements through using “drug libraries” (pre-loaded lists of medications and fluids), some participants expressed concerns about these developments: “*I think we all do mls per hour, and it’s probably safer to do that, really, because if you’ve got them programming it for milligrams, it would probably cause confusion, I think, to be honest. I think you can do that with them, but I’ve never… I don’t want to learn that way*” (Participant Q, ITU). Though smart pumps seem like a way to reduce error, the concerns expressed suggest that they could create a mismatch between the functionality provided by the pump and current nursing practice.

### Interface symbols and options

The device interface consists of a small display screen that includes: four arrow keys (up, down, left, right to navigate menus and input values); seven other buttons, including a yellow one labelled “BOL” (short for “bolus”—explained below) and a blue one with three white arrow-like symbols on it (to connect the pump to an additional programming station known as “Space Control”); and three LED lights: yellow, green/red and blue (to signify different alarms and statuses). Participants used all of the buttons, apart from “BOL” (as its function differed according to context—explained below) and the “Space Control” function (which was not required within the hospital). With respect to the latter, they were also unaware of what the function was for, e.g. “*That’s a good question. I’ve never used it*” (Participant P, ITU).

With respect to the display screen there was also some confusion over an option that appears after inserting the giving set into the pump and closing the door where the display prompts the user to select “SpaceLine”. This refers to the type of giving set being used where “SpaceLine” is the standard line (in oncology for instance they use a non-PVC set that does not react with chemotherapy drugs). While several participants were aware of what the term referred to e.g. “*So then it says, SpaceLine. That’s just the type of line it is*”. (Participant H, Paediatrics), others were not so sure e.g. when asked what the option was: “*It’s so that it starts, like, going into the line, I think* *”* (Participant N, Paediatrics).

In addition, there was some confusion over the various symbols displayed on the screen. Most participants were aware of the symbol that indicated the infusion had started running (moving arrows) and those that represented the battery supply and whether the device was plugged in, but there were additional symbols e.g. Therapy Profile (represented by an abstract symbol), which no one could explain, and presumably were not set up for use. The symbols relating to pressure were more commonly referred to, with some participants indicating a good understanding about what they represented e.g. “*Sometimes the line’s kinked, you can just straighten and that will do it. Shall I show you an occlusion? This is it. This is your pumping pressure*<kinks line and show us how the symbol changes>*”* (Participant Q, ITU). Depending on the clinical area, pressure options may or may not be configurable by the user. Pressure seemed a particular concern within Paediatrics due to the fact children tend to have smaller veins e.g. “*Our paediatric pumps are set to different pressures.* *So, like, the dotted line means the maximum pressure* *…* *So it’s not going to alarm here, but if it goes over that line*…” (Participant N, Paediatrics).

In general however, there was an assumption that the device itself would let them know whether there was a problem so they often did not pay attention to the majority of symbols. For instance, Participant K (Research) thought one of the pressure symbols related to the rate but went on to suggest “*we don’t usually pay attention to all that, we just deliver what needs to be delivered, not the technicalities of the machine*”. Similarly, Participant O (Surgery) suggested that the device would let them know whether something was wrong: “*You look at the pump to make sure that the pump is running …. But if the pump is, if there is any issue going on, like I said, the pump will be alarming anyway*”. The mismatches regarding the interface symbols and options are relatively minor given that the information ignored was not considered necessary for day-to-day activities. However, the issues do suggest a particular reliance on the pump to signify a problem, rather than ensuring a comprehensive examination of the patient and access points occurs. For instance, extravasation can occur when IV medications accidentally leak into the tissue around the infusion site but this is not something a pump is able to sense (Quinn 2008). Thus, there could be mismatch between what the user understands the pump is capable of and what it is actually able to do.

### Configuration of the device

While the same large volume pump was used across the hospital, there were differences between how devices in particular areas were configured in terms of the functions made available, even though the interface was the same. For instance, in Paediatrics, nurses were able to change pressure settings (but this feature was not available in other areas), while in the majority of clinical areas the pump was set up to be primed by pressing the “BOL” button (apart from in ITU). “BOL” is short for bolus: a single large dose of a medication that is given to a patient over a relatively short period of time. However, “priming” refers to the process of running the fluids from an intravenous bag through the giving set to make sure there is no air in the tubing before it is connected to the patient. While the mechanism the device uses to perform either task may the same (i.e. a programme that runs a certain volume of fluid through the pump relatively quickly), there was some confusion around these important clinical terms. For instance, Participant E (Research) stated: “*that’s what they were taught, the different way of bolusing it through the line, yes. I was talking to one of the senior chemo nurses, about priming the line and she says normally what she does is puts ten mls into the machine and does it manually* *…* *So, even she doesn’t know that the bolus line works*” while Participant M (Paediatrics) suggested “*This is the priming. It’s primed for the bolus*”. Thus, there is a mismatch between how nurses understand the concept of priming and how the pump represents this concept to users. However, as Participant P (ITU) explains “*…priming is different from bolus. Totally different. And in ITU we don’t… we stick to our priming and bolusing as separate, we don’t want to bring these things together and cause confusion*”.

In ITU the pump prompts the user to prime when at the start, and the “BOL” function is only used to programme a bolus dose. Presumably the decision was made to use the “BOL” button for priming in other areas of the hospital as the pump was not meant to be used for delivering a bolus outside intensive care. While participants were generally very aware of the importance of making sure that air bubbles did not reach a patient, these examples suggest how a mismatch can occur due to the way in which technology is set up (in this case, by the Medical Engineering Department), leading to a potentially confusing use of terms that are widely used within clinical practice.

### Checking

The Nursing and Midwifery Council’s Code of Professional Conduct states that nurses “are personally responsible for their practice” (NMC, 2004) where they are subject to professional and legal accountability if an error occurs (Hyde 2008). Thus, nurses are keen to minimise potential risks through ensuring appropriate checks have occurred prior to starting an infusion. As Participant L (Paediatrics) describes: “*You check that it was prescribed properly, you’ve got the right patient, that the right amount is going into the bag that you’re using, the right medicine, it’s in date …You’re checking all of those things, and then you’re checking the rate and the volume that is being entered*”. The process often involved checking the details of supporting artefacts as well as consulting a second person before administration occurs. However, while the majority of participants referred to the second checking of calculations and details such as the medication and dose to be delivered, it was not always clear exactly when these checks occurred and what was checked. For instance, some noted that second checking did not always extend to the values that had been entered into the pump before the infusion was started. For instance, Participant A (Oncology) notes: “*Ideally, we’re supposed to set the machine together. We’re supposed to confirm the rate. We don’t always do that, you’re so busy*”. In addition, Participant S (ITU) explains “*What doesn’t happen though, is, as that’s then administered to the patient, the same person isn’t called back to say, this is what I’m giving, this is the rate I’m giving it at … I’m not sure how it would work from a practical point of view, because that person that’s checked that drug with you could well be in a side room*”. So while second checking was seen as important, practical factors, such as demands on time and layout of the ward, can get in the way of getting another nurse to come back and check the device. Second checking by another person is not something the device explicitly supports, so there is a potential mismatch between the functionality of device and nursing practice.

There were instances however where participants would describe using the pump itself as a kind of second check for the values they entered into it. The device requires that the user put in two out of three values (VTBI, time or rate) and will calculate the third automatically. As Participant F (Oncology) explains: “*it’s very simple and straightforward, you put your volume, you put your hours and they calculate it for you. Compared to the previous pump that we used where we had to calculate the infusion rate and then put it manually into the system, this one can just put, either you just put your time that you want it to run over and it just calculates a certain rate or you put it at a certain rate and it gives you a certain time*”. In some cases nurses would rely on this to make sure they had put in the correct values e.g. “*I’m satisfied because I know it’s a half an hour infusion. So, if it said an hour I’d know I’ve got one of these two incorrect and I go back*” (Participant E, Research). However, using this pump in this way relied on an accurate understanding of the relationship between VTBI, time and rate, which not everyone exhibited (see below).

### Relationship between VTBI, time and rate

In the programming demonstrations (second interview), two participants showed confusion concerning the relationship between the three values that could be entered into the pump. In the first example, Participant O (Surgery) is demonstrating a particular infusion that requires an initial volume to be delivered over 15 min, where, if the patient does not exhibit an adverse reaction, the infusion will be continued over a longer period of time. However, during the process, the nurse mixes up two of the values and is surprised by what results. First, she enters the rate (which is supposed to be 20 ml per hour) as the VTBI: “*So 20 ml. Hold on. Volume to be infused. Okay. So 20 ml in an hour. And you want it for 15 min*”. However, once the user entered VTBI as 20 ml and time as 15 min, the pump automatically calculated a rate of 80 ml per hour. The nurse did not realise that the pump was set up with the incorrect rate until one of the interviewers pointed it out:

*Participant O*: *[referring to the rate] No, it’s 80 per hour. We’re just using 15* *min*.
*Interviewer I*: *Yes, but no, [Interviewer J] is right. So it’s 20* *ml per hour*.
*Participant O*: *Oh right. Oh goodness me, no*.

After some discussion and starting the process over, the nurse works out what the correct values should be: “*So the rate would be 20 ml in an hour, that’s right. And then 15 min will be just 5 ml to be infused*”. In this case the rate should have been 20 ml per hour, delivered in a time span of 15 min, leading to just 5 ml being delivered. If the pump had been connected to the patient and the correction had not been made, then the patient would have received 20 mils in 15 min, which is four times what this particular step in the drug protocol required.

In another example, Participant M (Paediatrics) has already entered a VTBI of 100 mls into the machine and a time of 30 min but is surprised by how high the rate is: “*but basically the rates keep coming up as high rates, which is 200…*”. After spending a few minutes going through the interface menu to check the values on the pump, the nurse reduces the rate to 50 mls and the duration automatically changed to 2 h: “*Let me just put 50 …. Okay, so it’s running now for two hours, which we’re supposed to run for half an hour*”. However, the nurse does not seem to realise that a much lower volume will be delivered to the patient within half an hour as a result (25 mls instead of the intended 100 mls).

While in both cases there may be additional factors to take into account (for Participant O the infusion demonstrated was not a routine one, while Participant M had just finished a night shift), it was clear from these examples that there is a mismatch between user conceptions and the machine interface, as the pump does little to support an understanding of how the concepts of VTBI, time and rate relate to each other.

### VTBI values

VTBI refers to the amount of fluid that the device is programmed to deliver to the patient, and is not necessarily the same as the total volume of the bag or bottle attached to the pump or the total volume of fluid a patient will receive as part of their treatment. Participants used the term frequently, though sometimes shortened it to volume, e.g. “*the same volume over 30 min*” (Participant C, Oncology). In addition, it can be important to track a patient’s fluid balance (their intake and output) e.g. for patients who are dehydrated. In order to track this, nurses have to navigate through the pump menu options and note down how much volume has been delivered to the patient at particular intervals e.g. “*So every hour you would see how much had gone through*” (Participant K, Paediatrics).

However, while the VTBI value that was entered into the pump could be the same as the amount specified on the administration chart or the amount listed on the infusion bag or bottle, this was not always the case (note that VTBI is not always specified, as in the case of continuous infusions that are delivered over several hours until a clinician decides to stop or change the prescription). The actual value entered depends on how a nurse conceptualises the infusion process and what type of infusion is being delivered. For instance, Participant B (Oncology) notes that, when administering chemotherapy, which involved additional medication being added to the bag: “*there’s always extra in the bag … so I 100 % know that there is at least 500, and there is 30 mls of drug. I put 530, that’s my volume. And then I, myself, would do it over an hour and 25 min, because that gives you five minutes for your flush. So you’re actually doing it in an hour and a half*”. A flush is an amount of solution delivered to make sure all the medication has been delivered to a patient. In the case of chemotherapy, Participant B explains: “*We have to finish the bag. Every drop of it. But then we have to flush it. So if you now have a nurse that puts it 500 mls, and there is 580, they actually don’t know how much is in the bag most of the time. It’s a guesstimate. So then the pump beeps, 500′s over. The patient says my drug’s done. You look in the bag, and it’s not done, so then first of all, now you have to tell the patient it’s not finished, so they get annoyed … and it’s a waste of time*”.

In contrast, some participants who made up an infusion themselves explained how they took out a particular amount of fluid and replaced it with the additional medication in order to keep the VTBI the same e.g. “*You would take out the 31.4 ml and put back in the 31.4 ml of the drug*” (Participant L, Paediatrics). In addition, others explained they would enter a lower amount for VTBI, particularly in relation to continuous infusions where they want to make sure that air does not enter the giving set. The concern was less about air reaching the patient (which the pump was supposed to detect and stop) and more about managing workloads with respect to trying to avoid spending more time setting up a new infusion rather than just spiking a new bag. As Participant A (Oncology) explains when carrying out a blood transfusion: “*So I would… for the first unit, I would deduct about 30 ml, because I’ve used 20 to prime the line and I need a bit left if I want it all to go through. I would do the same if… if it’s just the one unit, I would just put exactly the amount of volume*”. Similarly, Participant H (Paediatrics) notes: “*So they’ll write the fluid and they’ll put 500 ml, so that’s a 500 ml bag and they want that given at, like the one I just did, at 63 ml an hour … so we set it at, say, 480 or 470 because then what will happen is then the machine will alarm while there’s still a bit of fluid, so it’s not running through the pump dry … I think they automatically cut out before they give [air] to the patient, but it just stops it emptying and it stops the line emptying, because then if they’re continuing on fluids and they’re the same fluids, you can just then, if they’re prescribed, get another bag and connect them back up*”.

There are particular safety issues that nurses appear to be trying to avoid through giving themselves more time to set up the next infusion. For instance, Participant S (ITU) describes an incident where the VTBI entered meant that a bag of medication ran out before the nurse was able to set up a new one:

*Participant S*: *There has been an incident where there was inotropes in a bag and the volume to be infused was incorrect; the bag ran out, and the patient actually had a cardiac arrest*.
*Interviewer I*: *Really?*
*Participant S*: *Yes. The bag ran out, and by the time… and obviously the patient was receiving quite a considerable amount of the drug, so blood pressure walloped, and, yes*.
*Interviewer J*: *But was the alarm not noticed, or…? Because presumably the pump would alarm when it’s finished?*.
*Participant S*: *Yes…., no, it was…. but the time that it was…that it then took to reconstitute another bag, and I think…*.
*Interviewer I*: *Ah, ok, so the issue wasn’t, let’s say, air got to the patient, it was that they weren’t getting enough of the drug over a consistent period of time*.
*Participant S*: *Yes, yes*.

Part of the issue concerning the VTBI value appears to stem from an uncertainty about when exactly the device issues a “pre-alarm” to signal when the infusion is nearing completion. For example, when asked when the pre-alarm sounds, Participant S (ITU) answered: “*I don’t know, actually, when that alarm kicks in …. I don’t know how they’re programmed to alarm—the only thing, again, from experience, if I’m setting a pump up, I’ll always put the volume to be infused less than I know the volume is … I then basically obviously pick when the alarm alarms*”. Similarly, Participant R (ITU) suggested that “*I don’t actually know if they’re set specifically … it really is determined on how quickly the infusion is running at*” while Participant B (Oncology) explained: “*I don’t know what the time was, they had a pre-alarm, and I had them shortened. Because what happened was, is that they would sit there and beep for 10 min. And it was driving everybody crazy*”. Again, there seems to be a mismatch here in relation to the time nurses require to set up an infusion and when the pump is set to alarm to notify users an infusion is almost finished. This mismatch adds to the issues described above regarding the effect alarms have on people within the environment and the different ways nurses conceptualise the volume of fluid within a bag and giving set.

## Discussion

In this article we focus on exploring conceptions of infusion device practice and potential mismatches between user and device. Through understanding the interaction between external resources (the device interface and supporting artefacts) and internal resources (user conceptions) the findings revealed the ways in which user actions depend on the resources that are available to them. As Klein (1998) argues, much of behaviour is skilled, unproblematic and successful, particularly for experts, as in the examples of nurses using the pump to check certain calculations or entering a slightly different VTBI value than what was prescribed. In these cases, nurses are able to draw upon resources in a way that supports their day-to-day practice. However, potential issues occur when nurses are unable to do so, whether this is due to the way the device has been designed (e.g. different interface symbols) or configured (e.g. in the case of the “BOL” function), their own understanding based on how they have been trained and their own experience (e.g. of the relationship between VTBI, time and rate) and nursing practice (e.g. the use of different measurement units or whether second checking by another user is expected to occur or not).

Previous work has investigated mismatches in relation to a range of technologies including aviation (Baxter et al. 2007) and work systems (Attfield and Blandford 2011); our work illustrates how medical technologies can also be examined in terms of conceptual fit. Our approach involved adopting a combination of interview and teach-back, where we were able to elicit key user conceptions and gain insight into mismatches between user and device without interrupting user activities. By focusing on the key concepts users work with, our findings indicate how practices can vary, even within a single institution, and reveal potential mismatches that could lead to error and affect patient safety. We discuss the implications of our findings in relation to infusion device design and training below.

### Implications for design and training

With respect to the design of infusion device interfaces, previous research has focused on issues such as complex menu structures (Nunnally et al. 2004) or comparing a modified version of an interface with an existing one (Garmer et al. 2002). Instead, we focused on how well users understand different aspects of the interface. The findings highlight which functions and symbols were redundant (due to features not used within the institution) and which were problematic e.g. the fact that some users were unaware that “SpaceLine” referred to a type of giving set. In some areas more than one giving set could be used e.g. for blood or chemotherapy treatments, requiring a different option on the pump to be selected to ensure the correct settings were in place (such as particular pressure sensitivities required for different types of fluid). While SpaceLine was the only option in many cases and could generally be ignored, the fact that some nurses were selecting options they did not understand is problematic. Thus, there is potential to clarify the text options on the pump to make clear that the selections offered relate to different types of giving set, or even removing this step for pumps used in environments when only one option is available. In addition, an explanation of what the different symbols mean and which are most relevant to practice could be incorporated into device training sessions.

The findings of our study also illustrate how mismatches between user and device can result from decisions made by clinical engineering departments about how to configure the device in the first place. While a policy decision may have been made to disable the bolus function in areas outside ITU, it is not clear why it was also decided that the “BOL” button be used to prime instead; especially since given that the device can automatically prompt the user to “Prime the line” when starting a new infusion. Through adopting a human factors approach to understanding technology use within a wider system (Holden 2011), our findings not only reveal variations in practice regarding whether nurses prefer to prime the line manually or through the pump, but also indicate policy variations between clinical areas. Furthermore, this decision seems to have influenced how nurses refer to these terms, indicating that some have confused these important clinical concepts. To reduce this potential confusion, the device could be set up in all areas to prompt the user to “Prime the line” at the start of a new infusion, where the “BOL” button is either disabled completely or reserved only for carrying out a bolus infusion.

Checking is another issue where there is potential for error, particularly since it is not clear whether the values entered into the pump are always checked by a second person. Unfortunately, as previous research indicates (Armitage 2008), due to issues such as deference to authority, reduction of individual responsibility, shallow checks and lack of time, double-checking is not an assured way to reduce error. Furthermore, infusion devices do not explicitly support this aspect of practice, leading to attempted solutions such as using clocks on drip stands to display when checks need to occur (Iacovides, Cox and Blandford 2013). While independent (rather than side-by-side) checks (Armitage 2008) and advances in technology (such as in-built dose calculators) may help, there is further scope here to explore how infusion devices could support users in carrying out additional checks. However, while some participants showed their expertise by taking advantage of the fact that the device performs automatic calculations of a third variable after the first two are entered, this was not a widespread strategy. Using the device in this way would be a quick and easy way for individuals to check their own calculations, and is something that should be referred to explicitly within training to reinforce user understanding of the relationship between the three variables.

In addition, part of the reason why only some staff adopted this strategy may be that the device itself does not seem to support an understanding of the relationship between VTBI, time and rate. The display screen on the pump is relatively small, and when entering a particular value, only that value can be viewed on the screen so there is little indication that one of the others may change. A failure to appreciate the relationship between these values could lead to situations where a patient does not receive an infusion at the correct rate or within the appropriate time frame, potentially jeopardising their safety. In terms of design implications, this suggests that infusion devices would benefit from incorporating larger screens that are able to display a graphical representation of these concepts, making clear how they influence each other (a similar suggestion has previously been made by Nemeth 2009a, b) and thus reducing potential for confusion.

Another one of our findings illustrates that the VTBI value entered into the pump differed from what was prescribed due to factors such as how nurses conceptualised the amount of fluid in the bag or bottle and what type of infusion is being delivered. Previous work which examined the numbers used in a hospital via infusion pump logs has suggested that numbers such as 100, 500 and 1000 should have dedicated buttons in order to speed up the programming process (Wiseman et al. 2013). In contrast, our findings are that practice around what values are entered into the pump varies and that such dedicated buttons may be less useful. Thus, we would argue that, for this particular context at least, an infusion device with dedicated value buttons would be inappropriate and should be avoided.

### Further work

One of the limitations of our work is that we focused on a single institution and infusion device. While we attempted to improve the generalisability of our findings by including participants from across clinical areas, further work is required to examine how infusion device practice and associated conceptions vary within and across institutions. Though our findings would be useful to inform this particular institution’s training programme and how the device is set up across clinical areas, suggestions regarding design implications for infusion pumps in general would benefit from further work that considers the match between user and device in other hospital contexts.

In addition, while our analysis indicates that the range of medication units involved in preparing and administering infusions creates potential for error, the solution is not as simple as redesigning pumps to ensure that the units prescribed can be entered straight into the pump. Not only do prescribing practices vary between clinical areas, but the units depend on what type of infusion is being prescribed. In addition, nursing practice stems from how nurses are trained to deliver infusions and what they observe in the ward, which would also be hard to change. While recent developments in smart pump technology are attempting to address some of these issues, e.g. by providing dose calculators as part of the device software, this technology has yet to become widespread in the UK and its impact on safety is unclear (Iacovides et al. 2014). Further work needs to be carried out to examine smart pumps to assess whether the additional functions they provide, and how they are implemented, are effective in ensuring a “tighter” conceptual fit and reducing the chances of error occurring. An ongoing project (Blandford et al. 2016) is investigating this further.

This study has, on a small scale, highlighted the variability in practices in the ways that IV medications are prescribed and administered, pumps are configured, and staff are trained. While some of this variability is unavoidable, much of it is a consequence of evolving local practices over time (Wenger 1998). The findings highlight the need for a much larger programme of research into the systems and practices of IV medication management, with a view to standardising and optimising procedures and practices so as to better align the design and configuration of technology and the training of staff with the broader systems of practice they support.

### Conclusion

Within the context of medical device safety, Blandford, Vincent and Furniss (Blandford et al. 2014b) argue that “greater attention needs to be paid to learning points in actual use and user experience (i.e. work as done)” (p. 107) emphasising the need to “raise questions about the suitability of the design itself, and whether design and use are misaligned” to establish a device’s “fitness for purpose” (p. 108). Due to practical limitations, we were unable to carry out the interviews within the work environment of participants. However, by using real-world examples of prescribed IV infusions as part of the teach-back activity we were able to gain insights into work as done by tapping into how users conceptualised different aspects of practice around device use. In addition, by focusing on mismatches, we were able to consider aspects of device design and how the device has been implemented within different contexts, thus raising questions about its fitness for purpose. By making visible under-explored aspects of practice, our findings highlight ways in which users draw upon a range of resources in their day-to-day activities and indicate how vulnerabilities in the wider system can be created when users are unable to draw upon these resources successfully. In addition, such an approach can also be used to develop recommendations for design and training, for improving patient safety.
